# Binder-Free Electrode based on Electrospun-Fiber for Li Ion Batteries via a Simple Rolling Formation

**DOI:** 10.1186/s11671-020-03369-y

**Published:** 2020-07-13

**Authors:** Yuqiong Kang, Changjian Deng, Xinyi Liu, Zheng Liang, Tao Li, Quan Hu, Yun Zhao

**Affiliations:** 1 Shenzhen Key Laboratory on Power Battery Safety Research and Shenzhen Geim Graphene Center, Tsinghua Shenzhen International Graduate School, Shenzhen, 518055 China; 2 Hoffmann Institute of Advanced Materials, Shenzhen Polytechnic, Shenzhen, 518055 China; 3 Department of Chemistry and Biochemistry, Northern Illinois University, DeKalb, IL 60115 USA; 4 Department of Materials Science and Engineering, Stanford University, Stanford, CA 94305 USA; 5 Changsha Nanoapparatus Co., Ltd, Changsha, 410017 China

**Keywords:** Binder-free electrodes, Rolling press, High stability, Lithium ion batteries

## Abstract

With the demand for higher energy density and smaller size lithium-ion batteries (LIBs), the development of high specific capacity active materials and the reduction of the usage of inactive materials are the main directions. Herein, a universal method is developed for binder-free electrodes for excellent stable LIBs by rolling the electrospun membrane directly onto the commercial current collector. The rolling process only makes the fiber web denser without changing the fiber structure, and the fiber web still maintains a porous structure. This strategy significantly improves the structural stability of the membrane compared to the direct carbonized electrospun membrane. Moreover, this method is suitable for a variety of polymerizable adhesive polymers, and each polymer can be composited with different polymers, inorganic salts, etc. The electrode prepared by this method can be stably cycled for more than 2000 cycles at a current density of 2500 mA g^−1^. This study provides a cost-effective and versatile strategy to design the LIB electrode with high energy density and stability for experimental research and practical application.

## Background

Lithium-ion batteries (LIBs) are widely applied in portable devices, electric vehicles, and stationary energy storage systems [1, 2]. Energy density is one of the most important parameters for LIBs. Though much effort has been made to improve the specific capacity of the anode and/or cathode materials, the research of reducing the electrochemically inactive component in the electrode materials is limited. State-of-the-art battery preparation process with ~ 10 wt.% polyvinylidene fluoride (PVDF) and carbon materials as the binder and conductive additives, respectively, limits the specific capacity and energy density of LIBs [3]. The reduction of the amount of inactive materials in the electrode is an effective method to improve energy density. Therefore, the binder-free electrode, which only consists of active materials and conductive substrate, offers a new opportunity to enhance the energy density of electrodes [4].

Nowadays, the methods to prepare the binder-free electrode are mostly hydrothermal synthesis, vapor deposition, etc. [5–8], which operate generally under harsh conditions in a limited scale. Although binder-free electrodes can be easily fabricated by electrospinning technique with a simple, versatile, and cost-effective way [8], the as-prepared membranes often become brittle after carbonization [9]; thus, the electrodes have to be prepared by mixing and grinding the carbonized materials with PVDF in organic solution, which is not only time-consuming but also inefficient. The grinding process could lead to the decrease of particle size, the increase of surface area, and the exposure of active materials to the electrolyte, all of which will result in poor electrochemical performance [10]. Therefore, it is extremely important to design the stable electrospun membrane for advanced binder-free electrodes.

Here, a universal method is developed for binder-free electrodes for stable LIBs by rolling the electrospun membrane directly onto the commercial current collector. The porous structure of the fiber network can be maintained after the rolling process. This method significantly improves the structural stability of the membrane compared to the direct carbonized membrane. The power and energy density of the active materials can be significantly enhanced by the unique binder-free process. Besides, a variety of polymerizable adhesive polymers can be used as the electrospun membrane sources for this study, and inorganic salts or particles can be added into the polymers to fabricate high performance electrodes. The electrode prepared by this method can be stably cycled for more than 2000 cycles at a current density of 2500 mA g^−1^.

## Presentation of the Hypothesis

**Fig. 1:**
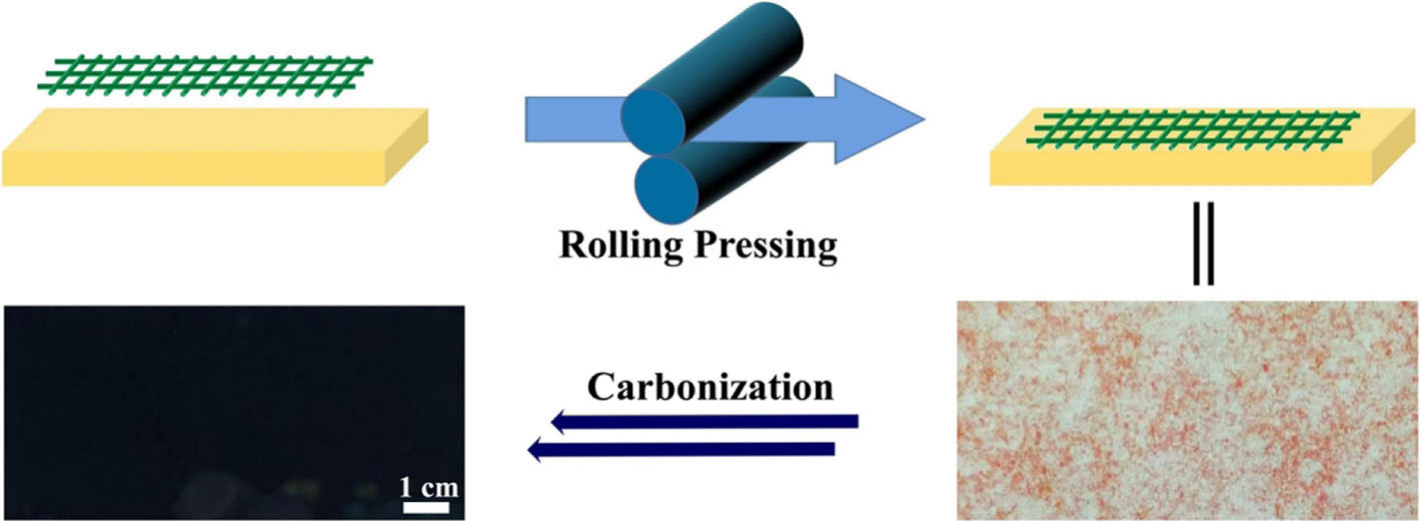
Schematic illustration of the fabrication of binder-free electrodes. The electrospun membrane is firstly pressed onto the current collector, then thermal treatment to achieve electrodes

Binder-free electrode is promising for lithium ion batteries with high energy density. A universal rolling press method is developed for binder-free electrodes for stable LIBs by rolling the electrospun membrane directly onto the commercial current collector. The porous structure of the fiber network can be maintained after the rolling process. This method improves the structural stability of the membrane compared to the direct carbonized membrane (Fig. 1).

### Testing the Hypothesis

#### Fabrication of Fiber Membranes

The coaxial electrospinning needles were purchased from Changsha Nanoapparatus China. The core-shell fiber membranes were obtained by extruding 10 wt.% polyacrylonitrile (PAN) and 8 wt.% polymethyl methacrylate (PMMA) in dimethylformamide (DMF) from outer and inner capillary, respectively. The flow rates of PAN and PMMA solutions were 0.54 and 0.27 mL h^−1^, respectively. A cylindrical roller covered with copper foil was placed vertically below the needle with a distance of about 11 cm to collect the fibers. The voltage was controlled at 14 kV. The obtained material was labeled as PMMA@PAN and PMMA@PAN@Cu after thermal treatment without and with Cu foil, respectively. The obtained membrane was firstly pressed by rolling press, then oxidized in air at 280 °C for 2 h with a heating rate of 5 °C min^−1^. Afterwards, it was moved to a tube furnace and carbonized at 650 °C for 2 h under flowing N_2_. The oxides@PMMA@PAN and oxides@PMMA@PAN@Cu were fabricated by the samemethod, where the inner solution of inorganic salts and PMMA and outer solution of PAN in DMF were extruded simultaneously.

#### Membrane Characterization

The morphology of the binder-free electrodes was characterized by scanning electron microscopy (SEM, Hitachi, SU-8010). The crystalline structure of the membranes was examined by X-ray diffraction (XRD, SmartLab, Rigaku) and Raman spectroscopy (Horiba, HR-800). XRD was tested with the 2θ between 5^o^ and 80^o^ under Cu Kα source (wavelength = 1.5406 Å). Raman spectroscopy was tested with an incident laser power of 100 mW from 1000 to 2000 cm^−1^.

#### Electrochemical Characterization

The electrochemical performance was evaluated using coin cells with fiber membrane discs as working electrode and lithium foil as the counter electrode. The electrolyte contained 1 mol L^−1^ LiPF_6_ in a mixture of ethylene carbonate (EC) and dimethyl carbonate (DMC) (v/v = 1:1). The galvanostatic discharge-charge cycling was examined in Land system (CT2001A, BTRBTS) in the voltage range of 0.01–3 V, and the current densities are set at 250 mA g^−1^ in the first 5 cycles for activation and gradually increased to 2500 mA g^−1^ in the following cycles.

## Implications of the Hypothesis

**Fig. 2:**
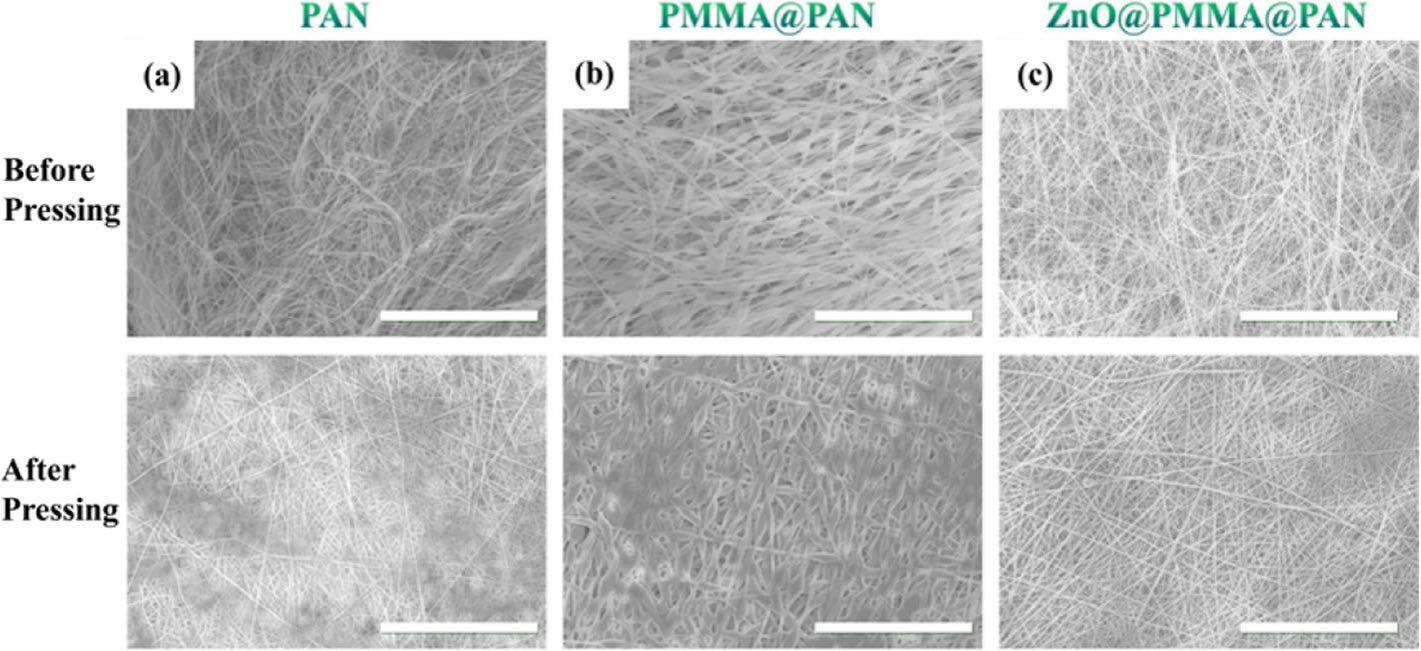
The morphology of electrospun membranes before and after pressing. **a** PAN. **b** PMMA@PAN. **c** ZnO@PMMA@PAN. Scale bars, 100 μm

Pressing process is just the physical combination of electrospun membrane and Cu foil. When pressing, the solvent-containing electrospun fibers are similar to the binder and adhere strongly to the current collector. The pressing process did not damage the porous structure of the materials (Fig. 2). After carbonized, the Cu foil will form a strong connection with the polymer. It is worth noting that this method is suitable for a variety of electrospun fibers, and here, three representative materials are demonstrated, namely, pure polymer (Fig. 2a), polymeric composite (Fig. 2b), and inorganic and polymeric composite (Fig. 2c).

**Fig. 3:**
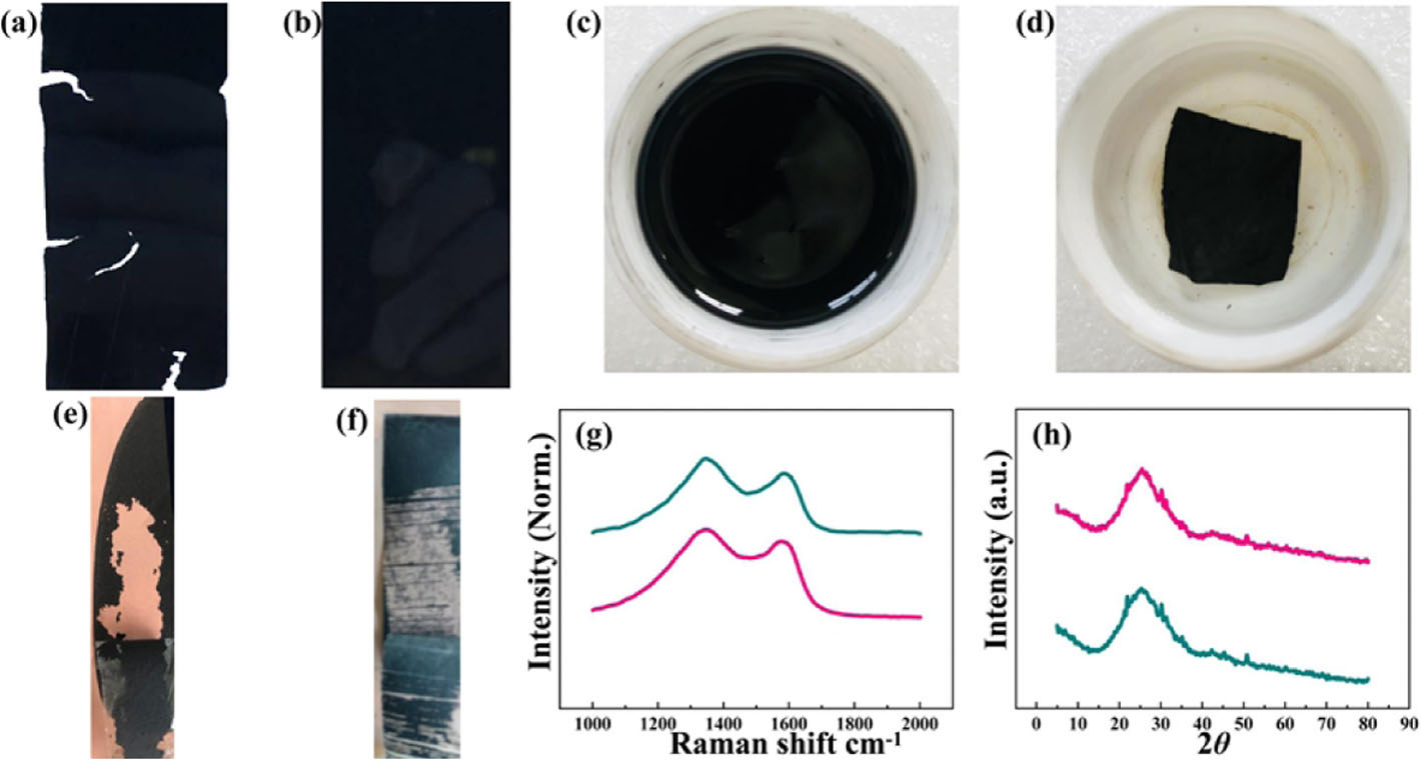
The characterizations of binder-free electrodes. Images of **a** PMMA@PAN and **b** PMMA@PAN@Cu. The stability of **c** PMMA@PAN and **d** PMMA@PAN@Cu after ultrasonic treatment for 30 min. Peeling test of **e** PMMA@PAN and **f** PMMA@PAN@Cu. **g** Raman and **h** XRD curves of PMMA@PAN and PMMA@PAN@Cu, respectively

PMMA@PAN membrane is selected as the example for the stability study of the carbonized membrane because the PAN membrane has relatively good film formation, while PMMA@PAN and oxides@PMMA@PAN membranes have poor stability and similar structures. As can be seen in Fig. 3a, PMMA@PAN membrane becomes brittle after carbonization, and cracks can be obviously observed. In contrast, the PMMA@PAN@Cu is very smooth with no cracks (Fig. 3b). This method enables the high-quality binder-free electrodes in large-scale production (about 5 cm × 10 cm) in the laboratory. To further demonstrate the structural stability of materials, the PMMA@PAN and PMMA@PAN@Cu are placed in ethanol solution for ultrasonic treatment for 30 min to test the strength of the membrane. It shows that PMMA@PAN starts to break at the beginning of the treatment and is completely destroyed and dispersed in ethanol after about 5 min, whereas the PMMA@PAN@Cu remains intact after 30 min where there are no visible cracks (Fig. 3c, b). Moreover, PMMA@PAN powder is ball-milled and coated onto the Cu foil with PVDF as binder to test the adhesion as shown in Fig. 3e. PMMA@PAN is easily aggregated during milling process. In addition, the surface of the fabricated electrode is quite rough, and the active materials can be entirely peeled. However, a large amount of PMMA@PAN@Cu material smoothly remains on the Cu foil after the same testing process (Fig. 3e, f). The ultrasonic treatment and adhesion test clearly demonstrate that the carbon material of the PMMA@PAN@Cu has a strong adhesion to the Cu foil [11].

The crystal structure of PMMA@PAN and PMMA@PAN@Cu is characterized by Raman spectroscopy and XRD to observe the differences after pressing the polymer fibers onto the Cu foil (Fig. 3 g, h). The first peak of Raman spectra at about 1350 cm^−1^ and the second at 1590 cm^−1^ corresponds to the D band of defect-induced mode and the G-band of E_2g_ graphitic mode, respectively [12]. The intensity ratios between the D and G band indicating the disorder degree of carbon materials. It shows the same value of 1.2 demonstrating the negligible impact after pressing the polymer fibers onto the Cu foil. Moreover, the disorder feature may be caused by the PMMA, which leads to the uneven carbonization of PAN and brittle property of the material. PMMA@PAN and PMMA@PAN@Cu have similar XRD pattern where both show strong diffraction peaks of 2θ value at 25.0^°^. This featured peak is corresponding to layers of the graphite structure [13]. In short, the carbonization process of the electrospun membrane has not changed after being composited with Cu foil.

### Electrochemical Performance

**Fig. 4:**
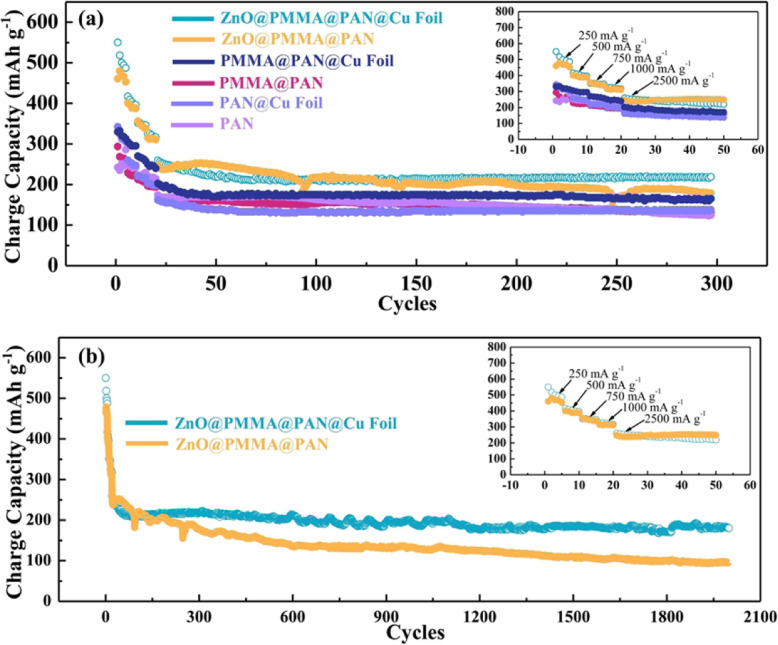
**a**, **b** Cycling performances of different binder-free electrodes, and the corresponding rate performances showed in the insert images

The electrochemical performances of various binder-free electrodes are examined using a CR2032 coin-type half-cells. The rate performances at current densities ranging from 250 to 2500 mA g^−1^ are displayed in Fig. 4a. The discharge capacity of ZnO@PMMA@PAN@Cu, ZnO@PMMA@PAN, PMMA@PAN@Cu, PMMA@PAN, PAN@Cu, and PAN can remain at 260, 248, 202, 163, 174, and 162 mAh g^−1^ at the current density of 2500 mA g^−1^, respectively. However, the capacity retention with the increasing of current density is generally lower after pressing the polymer fibers onto the Cu foil. It is mainly because that the pressed electrodes show less porosity, and some fibers are crushed together, limiting the Li ions transfer from electrolyte into the carbon materials. After 300 cycles, the discharge capacity remains at 219, 178, 165, 137, 130, and 124 mAh g^−1^ for ZnO@PMMA@PAN@Cu, ZnO@PMMA@PAN, PMMA@PAN@Cu, PMMA@PAN, PAN@Cu, and PAN, respectively. The capacity retention of the electrodes prepared by pressing the polymer fibers onto the Cu foil and carbonization keeps almost 100% from the 50th cycle while the membrane without Cu foil supporting show poor retention, namely, about 71%, 89%, and 81% for ZnO@PMMA@PAN, PMMA@PAN, and PAN, respectively. The cycle life of ZnO@PMMA@PAN@Cu and ZnO@PMMA@PAN is evaluated at a current density of 2500 mA g^−1^ (Fig. 4b). ZnO@PMMA@PAN@Cu and ZnO@PMMA@PAN show the reversible capacities of 180 and 96 mA h g^−1^ and the capacity retention of 82% and 55% after 2000 cycles, respectively. It demonstrates the excellent cycling performance after pressing the polymer fibers onto the Cu foil.

## Conclusions

A universal method is developed for binder-free electrodes for LIBs with stable electrochemical performance. This method is not only suitable for the preparation of binder-free electrodes, but also has the potential to be a current collector protection strategy. A thin layer of active carbon material can be coated on the surface of the current collector to avoid the contact of current collector and electrolyte without increasing the content of inactive materials. It is believed that not only Cu foil but also Al foil can achieve similar functions. In addition, the adhesion between the binder and the current collector can be enhanced by coating the carbon onto the current collector. Therefore, it is more convenient to develop high energy density electrode by utilizing this strategy.

## Data Availability

All data are fully available without restriction.
